# Synthesis of Some Novel Biologically Active Disperse Dyes Derived from 4-Methyl-2,6-dioxo-1-propyl-1,2,5,6-tetrahydro-pyridine-3-carbonitrile as Coupling Component and Their Colour Assessment on Polyester Fabrics

**DOI:** 10.3390/molecules17088822

**Published:** 2012-07-25

**Authors:** Samar M. Ashkar, Morsy A. El-Apasery, Marwan M. Touma, Mohamed H. Elnagdi

**Affiliations:** 1 Department of Chemistry, Faculty of Science, University of Aleppo, P.O. Box 10229, Aleppo, Syria; 2 Dyeing, Printing and Textile Auxiliaries Department, Textile Research Division, National Research Centre, Dokki, Giza 12622, Egypt; 3 Chemistry Department, Faculty of Science, Kuwait University, P.O. Box 5969, Safat 13060, Kuwait

**Keywords:** arylhydrazonopyridinones, biological activity, disperse dyes, polyester fabrics

## Abstract

A series of novel azo-disperse dyes containing alkylhydrazonopyridinone structures were synthesized. 4-Methyl-2,6-dioxo-1-propyl-1,2,5,6-tetrahydropyridine-3-carbonitrile (**8**) is synthesized by one-pot synthesis using ethyl cyanoacetate, propylamine, and ethyl acetoacetate. Compound **8** is then coupled with aromatic and heteroaromatic diazonium salts to afford the corresponding aryl- and heteroaryl-4-methyl-2,6-dioxo-1-propyl-1,2,5,6-tetrahydropyridine-3-carbonitriles **12a,b** and **13a–c**. Structural assignments to the dyes were made using NMR spectroscopic methods. A high temperature dyeing method was employed to apply these dyes to polyester fabrics. Most of the dyed fabrics tested displayed very good light fastness levels and good wash fastness. Finally, the biological activity of the prepared dyes against Gram positive bacteria and Gram negative bacteria were evaluated.

## 1. Introduction

Arylhydrazonopyridinones are now replacing arylazopyrazolones in the classical dye industry. Moreover, reasonable solubility of these derivatives in lipophilic solvents gives these dyes high potential for utilization in dye diffusion thermal transfer printing [1]. Pyridinone derivatives are relatively recent heterocyclic intermediates for the preparation of dyes. The azopyridinone dyes give bright hues and are therefore of investigative interest [2]. Owing to their improved brightness and light fastness, pyridinone disperse dye derivatives have found many applications on different fibres [3,4,5,6,7,8].

Moreover, the pyridinone nucleus has been proven to constitute the active part of several biologically active compounds [9,10]. Keeping in mind the biological importance of the above mentioned heterocyclic compounds and in continuation to our endeavor towards environmentally benign synthesis [11,12,13,14,15,16,17], we report herein the synthesis of 4-methyl-2,6-dioxo-1-propyl-1,2,5,6-tetrahydro-pyridine-3-carbonitrile (**8**) as a good precursor to novel aryl- and heteroaryl-4-methyl-2,6-dioxo-1-propyl-1,2,5,6-tetrahydropyridine-3-carbonitriles and their application as disperse dyes for the dyeing of polyester fabrics. The antibacterial activities of these dyes were also studied.

## 2. Results and Discussion

### 2.1. Synthesis and Characteristics

Our initial strategy aimed to synthesize the alkylhydrazonopyridinone **8** and convert it to the corresponding aryl- or heteroaryl-4-methyl-2,6-dioxo-1-propyl-1,2,5,6-tetrahydropyridine-3-carbo-nitriles **12a,b** and **13a–c**, which were anticipated to be excellent new disperse dyes for the dyeing of polyester fabrics employing a high temperature dyeing method. We introduce a one-pot three component condensation for the synthesis of alkylhydrazonopyridinone **8** under reflux and solvent-free conditions. Thus, the reaction of ethyl acetoacetate, ethyl cyanoacetate, and propyl amineunder reflux without solvent for 6 hours afforded compound **8** in good yield. The product was identified by IR and ^1^H-NMR spectroscopic data. Compound **8** may be exist in another tautmeric form **9** and in solution there is a very fast equilibrium between them [18,19]. The structure of compound **9** in solution was confirmed based on the basis of ^1^H-NMR spectral data (presence of a pyridonyl-H signal at δ 7.67 and hydroxyl group signal at δ 14.63, respectively).

**Scheme 1:**
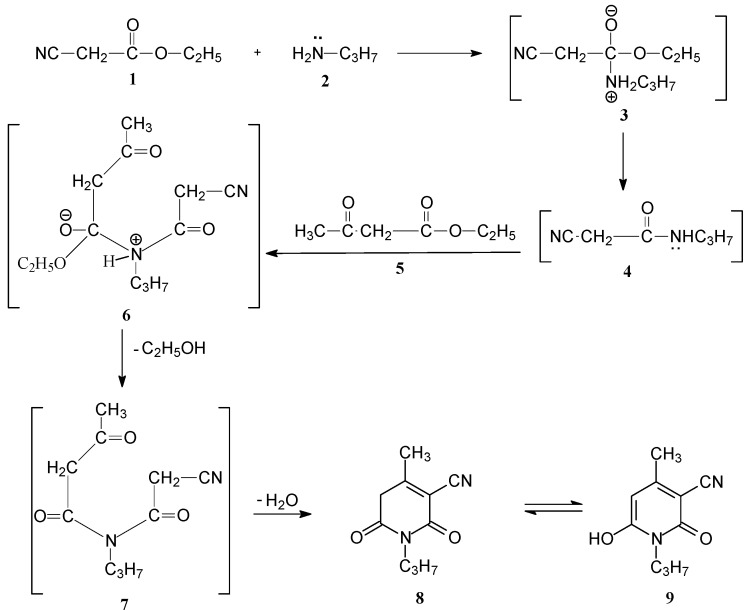
Preparation of 4-methyl-2,6-dioxo-1-propyl-1,2,5,6-tetrahydropyridine-3-carbonitrile (**8**).

It seems that the *in-situ* reaction of alkylcyanoester **1** and primary amine **2** leads to amide derivative **4** and reaction with *β*-ketoester **5** then gives 4-methyl-2,6-dioxo-1-propyl-1,2,5,6-tetrahydropyridine-3-carbonitrile (**8**) in high yield. The formation of compound **8** is assumed to proceed via intermediacy of **3**, **4**, **6** and **7**. The one-pot nature of the present procedure makes it an acceptable alternative to multistep approaches. It also simplifies the laborious procedures and offers considerable advantages over the existing methodologies, such as elimination of solvent, high yield, and environmentally friendly character (*cf.* Scheme 1).

**Scheme 2:**
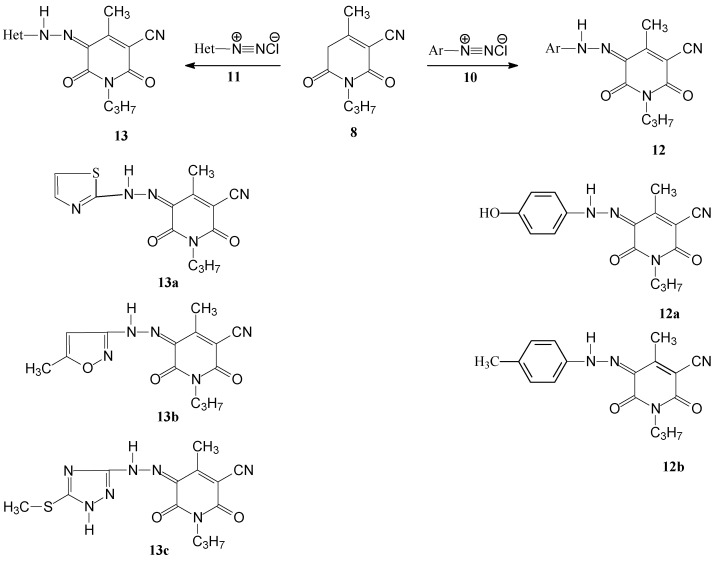
Synthesis of some novel biologically active disperse dyes **12a,b** and **13a–c**.

Coupling of compound **8** with aromatic and heteroaromatic diazonium salts (Ar and Het, respectively) afforded the corresponding aryl- and heteroaryl-4-methyl-2,6-dioxo-1-propyl-1,2,5,6-tetrahydropyridine-3-carbonitriles **12a,b** and **13a–c**. Structural assignments to these dyes were made using NMR spectroscopic methods (*cf.* Scheme 2).

### 2.2. Dyeing and Fastness Properties

Disperse dyes **12a,b** and **13a,b** were applied to polyester fabrics at 1% o.w.f., using high temperature dyeing method (HT) at 130 °C. Dark orange, yellow, orange and light green color shades were obtained. The dyeing properties on the polyester fabrics were evaluated in terms of their fastness properties (e.g., fastnesses to washing and light). The color of dyeing on polyester fabrics is expressed in terms of CIELAB values (Table 1), and the following CIELAB coordinates were measured: lightness (L*); chroma (C*); hue angle (H) from 0 to 360°; a*, whose value represents the degree of redness (positive) and greenness (negative); and b*, whose value represents the degree of yellowness (positive) and blueness (negative).

**Table 1:** Optical measurements of the synthesized dyes on the polyester fabrics.

Dye No.	DL*	Da*	Db*	DC*	Dh*	*CMC dE*
12a	−25.66	40.68	84.09	92.89	−9.82	126.14
12b	−14.95	7.24	97.52	97.63	−5.52	131.81
13a	−22.16	18.14	83.08	84.77	−6.75	114.79
13b	−13.22	−8.06	42.03	36.13	−0.37	48.89

In general, the color hues of the dye **13b** on the polyester fabric shifted in the greenish direction; this was indicated by the negative values of a* (red–green axis). The positive values of b* (yellow–blue axis) indicated that the color hues of the dyes **12a, 12band 13a** on the polyester fabric shifted to the yellowish direction.

The physical data for the dyed fibers, given in Table 2, shows that the disperse dyeing displayed good wash fastness properties to five multiple washes in terms of shade change and also very little staining to the adjacent fiber strip occurred. Table 2, shows that the light fastness is very good with respect to most of the tested compounds, except compound **13a** (1% o.w.f) which showed good results.

**Table 2:** Shade and Fastness properties of azo disperse dyes on polyester fabrics.

Dye No.	Color shade on polyester	Wash fastness *			Light fastness
		Washes	Change in colour	SC	
12a	Dark orange	1	4–5	5	7–8
		2	3–4	4	
		3	3–4	4	
		4	3–4	4	
		5	3–4	4	
12b	Yellow	1	4–5	5	7
		2	4–5	4–5	
		3	4	4-5	
		4	4	4–5	
		5	4	4–5	
13a	Orange	1	4–5	5	5–6
		2	4–5	4–5	
		3	4	4–5	
		4	4	4–5	
		5	4	4-5	
13b	Light green	1	4–5	5	6–7
		2	4	4	
		3	4	4	
		4	4	4	
		5	4	4	

* SC = staining on cotton.

### 2.3. Antimicrobial Activities

The antimicrobial activities of the synthesized dyes were screened against selected bacteria by the agar well diffusion method and their inhibition zones diameters, given in Table 3, reveal that three of the tested dyes showed positive antimicrobial activities against at least one of the tested microorganisms. Two dyes, **12a** and **13a** showed strong activities, while two dyes **13b** and **13c** showed moderate activities with significant inhibition zones >10 mm, against *Staphylococcus auerus* and *Bacillus subtilus* (Gram positive bacteria). It is worth mention here that dye **12b** has showed no antimicrobial activities against any of the tested microorganisms, and all the dyes have no antimicrobial activities against *Escherichia coli*, *Klebsiellae pneumoniae* and *Pseudomonas aeruginosa* (Gram negative bacteria).

**Table 3:** Diameter of the zones of inhibition of the tested compounds that showed weak to strong antimicrobial against microorganisms.

Dye No.	Conc. % of Dye	Inhibition zone diameter (Nearest mm)				
		Gram positive bacteria		Gram negative bacteria		
		*S. aureus*	*B. subtilus*	*E. coli*	*K. pneumonia*e	*P. aeruginosa*
12a	0.25 mg/mL	-	-	-	-	-
12b		-	-	-	-	-
13a		-	-	-	-	-
13b		-	-	-	-	-
13c		-	-	-	-	-
12a	0.50 mg/mL	-	-	-	-	-
12b		-	-	-	-	-
13a		16	16	-	-	-
13b		-	-	-	-	-
13c		-	-	-	-	-
12a	1.00 mg/mL	18	13	-	-	-
12b		-	-	-	-	-
13a		20	19	-	-	-
13b		-	14	-	-	-
13c		10	10	-	-	-
12a	2.00 mg/mL	21	18	-	-	-
12b		-	-	-	-	-
13a		24	20	-	-	-
13b		-	15	-	-	-
13c		12	12	-	-	-
12a	3.00 mg/mL	25	22	-	-	-
12b		-	-	-	-	-
13a		30	22	-	-	-
13b		-	17	-	-	-
13c		16	16	-	-	-

(-): No inhibition.

The results obtained for the synthesized compounds in this study support the findings of the other researchers [9] who showed that alkylhydrazonopyridinones cores have various biological activities. alkylhydrazonopyridinones were proved to possess biological effects, including antimicrobial activities [10]. Therefore, the new synthesized classes showed promising results for possessing the potentials to be utilized for medicinal purposes.

## 3. Experimental

### 3.1. General

Melting points were recorded on a Gallenkamp apparatus. IR spectra were recorded using KBr pellets on a Jasco FTIR-6300 FT-IR spectrophotometer. ^1^H and ^13^C-NMR spectra were recorded on Bruker DPX 400 MHz. The colorimetric parameters of the dyed polyester fibers were determined on a reflectance spectrophotometer (Datacolor SF 600X Spectraflash, Switzerland). Mass spectra were measured on a high resolution GC/MS DFS-Thermo. Microanalyses were performed on Elementar-Vario Micro cube Analyzer.

### 3.2. Synthesis of 4-Methyl-2,6-dioxo-1-propyl-1,2,5,6-tetrahydropyridine-3-carbonitrile *(8)*

A mixture of ethyl acetoacetate (26.00 g, 0.2 mol), ethyl cyanoacetate (22.62 g, 0.2 mol), propylamine (11.82 g, 0.2 mol) was refluxed until the reaction was completed (about 6 h). During the reaction, the white product precipitated. The crude product was filtered, dried and recrystallized from ethanol to give white crystals (82%), m.p. 218–220 °C, IR (KBr): 2217 (CN) and 1735 (CO) cm^−1^; ^1^H-NMR (400 MHz, DMSO-d_6_): δ = 0.85 (t, *J* = 7 Hz, 3H, CH_3_CH_2_), 1.49 (m, 2H, CH_2_), 2.39 (s, 3H, CH_3_), 3.69 (s, 2H, CH_2_N), 7.67 (s, 1H, CH), 14.63 (br, 1H, OH, D_2_O exchangeable); MS (EI): *m/z*(%) = 193 ([M+1]^+^, 56). 151 (12), 120 (5), 94 (7)*.*

### 3.3. General Procedure for the Synthesis of Azo Disperse Dyes

A cold solution of arenediazonium salt (10 mmol) was prepared by adding a solution of sodium nitrite (1.00 g in 10 mL H_2_O) to a cold solution of aryl amine hydrochloride or aryl amine nitrate (10 mmol) with stirring as described earlier. The resulting solution of the arenediazonium was then added to a cold solution of compound **8** (10 mmol) in ethanol (20 mL) containing sodium acetate (2.00 g). The mixture was stirred at room temperature for 1 h and the solid product so formed was collected by filtration and recrystallized from ethanol to give compounds **12a,b** and **13a–c**, respectively*.*

*5-[(4-Hydroxyphenyl)-hydrazono]-4-methyl-2,6-dioxo-1-propyl-1,2,5,6-tetrahydropyridine-3-carbo-nitrile* (**12a**). Red crystals from toluene (88%), m.p. 252–255 °C, IR (KBr): 3407 (OH), 2222 (CN) and 1657 (CO) cm^−1^; ^1^H-NMR (400 MHz, DMSO-d_6_): δ = 0.85 (t, *J* = 7 Hz, 3H, CH_3_CH_2_), 1.51 (m, 2H, CH_2_), 2.45 (s, 3H, CH_3_), 3.78 (t, *J* = 7 Hz, 2H, CH_2_N), 6.86 (d, *J* = 7 Hz, 2H, arom-H), 7.55 (d, *J* = 7 Hz, 2H, arom-H), 10.02 (br, 1H, OH, D_2_O exchangeable) 14.84 (br, 1H, NH, D_2_O exchangeable); ^13^C-NMR (DMSO-d_6_): δ = 160.5, 160.0 (2CO), 158.7, 157.5, 133.0, 121.5, 119.2, 116.3, 115.4, 98.2, 29.3 (CH_2_), 20.4 (CH_2_). 16.1 (CH_3_), 11.2 (CH_3_). MS (EI): *m/z* (%) = 312 ([M]^+^, 100). 295 (8), 270 (52), 121 (24), 108 (43), 93 (24). HRMS: *m/z* (EI) for C_16_H_16_N_4_O_3_; calcd. 312.1216; found: 312.1216.

*4-Methyl-2,6-dioxo-1-propyl-5-(p-tolylhydrazono)-1,2,5,6-tetrahydropyridine-3-carbonitrile* (**12b**). Yellow crystals from ethanol (86%), m.p. 215–218 °C, IR (KBr): 3432 (NH), 2223 (CN) and 1679 (CO) cm^−1^; ^1^H-NMR (400 MHz, DMSO-d_6_): δ = 0.88 (t, *J* = 7 Hz, 3H, CH_3_CH_2_), 1.52 (m, 2H, CH_2_), 2.32 (s, 3H, CH_3_), 2.50 (s, 3H, CH_3_), 3.78 (t, *J* = 7 Hz, 2H, CH_2_N), 7.27 (d, *J* = 7 Hz, 2H, arom-H), 7.58 (d, *J* = 7 Hz, 2H, arom-H), 14.62 (s, 1H, NH, D_2_O exchangeable); MS (EI): *m/z* (%) = 310 ([M]^+^, 100). 293 (9), 268 (26), 177 (12), 106 (32), 91 (56). HRMS: *m/z* (EI) for C_17_H_18_N_4_O_2_; calcd. 310.1424; found: 310.1424.

*4-Methyl-2,6-dioxo-1-propyl-5-(thiazol-2-yl-hydrazono)-1,2,5,6-tetrahydropyridine-3-carbonitrile* (**13a**). Yellow crystals from toluene (65%), m.p. 210 °C, IR (KBr): 3405 (NH), 2224 (CN) and 1684 (CO) cm^−1^; ^1^H-NMR (400 MHz, DMSO-d_6_): δ = 0.87 (t, *J* = 7 Hz, 3H, CH_3_CH_2_), 1.50 (m, 2H, CH_2_), 2.47 (s, 3H, CH_3_), 3.79 (t, *J* = 7 Hz, 2H, CH_2_N), 7.48 (d, *J* = 7 Hz, 1H, arom-H), 7.67 (d, *J* = 7 Hz, 1H, arom-H), MS (EI): *m/z*(%) = 303 ([M]^+^, 100). 288 (7), 218 (12), 190 (90), 149 (17), 86 (27). HRMS: *m/z*(EI) for C_13_H_13_N_5_O_2_S; calcd. 303.0782; found: 303.0782.

*4-Methyl-5-[(5-methylisoxazol-3-yl)-hydrazono]-2,6-dioxo-1-propyl-1,2,5,6-tetrahydropyridine-3-carbonitrile* (**13b**). Yellowish-green crystals from ethanol (80%), m.p. 266–268 °C, IR (KBr): 3429 (NH), 2225 (CN) and 1688 (CO) cm^−1^; ^1^H-NMR (400 MHz, DMSO-d_6_): δ = 0.88 (t, *J* = 7 Hz, 3H, CH_3_CH_2_), 1.51 (m, 2H, CH_2_), 2.44 (s, 3H, CH_3_), 2.45 (s, 3H, CH_3_), 3.79 (t, *J* = 7 Hz, 2H, CH_2_N), 6.67 (s, 1H, arom-H), 14.24, 14.62 (s, 1H, NH, D_2_O exchangeable); MS (EI): *m/z* (%) = 301 ([M]^+^, 100). 286 (8), 216 (52), 190 (72), 149 (42), 68 (42). HRMS: *m/z* (EI) for C_14_H_15_N_5_O_3_; calcd. 301.1163; found: 301.1163.

*4-Methyl-5-[(5-methylsulfanyl-1H-[1,2,4]triazol-3-yl)-hydrazono]-2,6-dioxo-1-propyl-1,2,5,6-tetra-hydropyridine-3-carbonitrile* (**13c**). Yellow crystals from ethanol (82%), m.p. 271–272 °C, IR (KBr): 3431 (NH), 2232 (CN) and 1684 (CO) cm^−1^; ^1^H-NMR (400 MHz, DMSO-d_6_): δ = 0.88 (t, J = 7 Hz, 3H, CH_3_CH_2_), 1.51 (m, 2H, CH_2_), 2.47 (s, 3H, CH_3_), 2.50 (s, 3H, CH_3_), 3.78 (t, J = 7 Hz, 2H, CH_2_N), 14.20 (s, 1H, NH), 14.60 (s, 1H, triazolyl-NH), MS (EI): m/z (%) = 333 ([M]^+^, 100). 291 (12), 256 (12), 205 (32), 149 (12), 97 (36). HRMS: m/z (EI) for C_13_H_15_N_7_O_2_S; calcd. 333.1002; found: 333.1002.

### 3.4. High Temperature Dyeing Method (HT)

#### 3.4.1. Materials

Scoured and bleached polyester 100% was used. The fabric was treated before dyeing with a solution containing non-ionic detergent (5 g/L) and sodium carbonate (2 g/L) in a ratio of 50:1 at 60 °C for 30 min, then thoroughly washed with water and air dried at room temperature.

#### 3.4.2. Dyeing

The dye baths were prepared from the dye (1.0% weight of fibre) to a final liquor of 40:1, w/w. The pH value of the bath was adjusted to 4–5 with acetic acid (10%). The polyester fabrics, previously wetted, were placed into the liquor at 25 °C–30 °C. The temperature was raised to 130 °C at the rate of 2 °C/min, and dyeing continued for 60 min. After cooling, the dyed fabrics were then washed and dried [20].

#### 3.4.3. Color Assessment

The colorimetric parameters (Table 1) of the dyed polyester fibers were determined on a reflectance spectrophotometer (Datacolor SF 600X) equipped with a D65 source.

### 3.5. Fastness Testing

The dyed fabrics were tested, employing ISO standard methods [21]. Wash fastness tests were carried out in accordance with ISO: 105-C01-C05:1989(E):Test 1–5, ISO: 105-A02:1993(E) and ISO: 105-A03:1993(E). Daylight fastness was carried out in accordance with ISO: 105-B01:1994.

### 3.6. Antimicrobial Activities Test

The antimicrobial activities of four disperse dyes were tested against five different microbial cultures using the agar-well diffusion technique [22]. Pure cultures of *Staphylococcus auerus* and *Bacillus subtilus* (Gram positive bacteria), *Escherichia coli*, *Klebsiellae pneumoniae* and *Pseudomonas aeruginosa* (Gram negative bacteria) were employed in the test. An aliquot of each bacterial strain (0.1 mL) was inoculated and spread on nutrient agar (NA). The inoculated plates were supplied with 100 µL of each of the tested dyes with concentrations of 0.25, 0.50, 1.00, 2.00 and 3.00 mg·mL^−1^. The dyes were placed in 4 mm wells produced by sterile cork borer. The NA plates were incubated at 37 °C for 24 h. The zones of inhibition around the wells were determined and the averages based on three replicas were recorded.

## 4. Conclusions

In summary, 4-methyl-2,6-dioxo-1-propyl-1,2,5,6-tetrahydropyridine-3-carbonitrile (**8**) was synthesized by one-pot synthesis using ethyl cyanoacetate, propylamine, and ethyl acetoacetate. Compound **8** is then coupled with aromatic and heteroaromatic diazonium salts to afford the corresponding aryl- and heteroaryl-4-methyl-2,6-dioxo-1-propyl-1,2,5,6-tetrahydropyridine-3-carbonitriles. The dyes produced in this manner were then applied to polyester fabrics by using a HT dyeing method. The dyed fabrics, which displayed yellow to light green hues on polyester fabrics, have very good fastness level to light and good fastness to wash. Finally, the biological activity of the synthesized dyes against Gram positive bacteria, Gram negative bacteria was discussed. 
